# The expanding role of artificial intelligence in personalised medicine: from innovation to individualized care

**DOI:** 10.3389/fmed.2026.1795879

**Published:** 2026-04-07

**Authors:** Abrar Ali, Mohd Adnan Kausar, Sadaf Anwar, Farida Habib Khan, Halima Mustafa Elagib, Mona M. Shahien, Fahad M. Alshabrmi

**Affiliations:** 1 Department of Ophthalmology, College of Medicine, University of Ha'il, Hail, Saudi Arabia; 2 Department of Biochemistry, College of Medicine, University of Ha'il, Hail, Saudi Arabia; 3 Department of Community and Family Medicine, College of Medicine, University of Ha'il, Hail, Saudi Arabia; 4 Department of Pharmacology, College of Medicine, University of Ha’il, Hail, Saudi Arabia; 5 Department of Pediatrics, College of Medicine, University of Ha'il, Hail, Saudi Arabia; 6 Department of Medical Laboratories, College of Applied Medical Sciences, Qassim University, Buraydah, Saudi Arabia

**Keywords:** artificial intelligence, deep learning, drug discovery, genomics, machine learning, precision medicine

## Abstract

Artificial intelligence (AI) has become a transformative force in precision medicine, enabling unprecedented personalization of healthcare therapies via sophisticated computational analysis of complex biomedical data. This review examines the evolving landscape of AI applications in precision medicine, highlighting both established technologies and emerging advances that are reshaping clinical practice. Current applications demonstrate the ability of AI to enhance diagnostic precision, refine treatment decisions, accelerate drug development, and improve patient outcomes across multiple disease domains. Despite significant progress, challenges related to data standardization, algorithmic bias, legal frameworks, and equi7 access remain key barriers to broader implementation. As technical capabilities advance and integration with healthcare systems deepens, AI-driven precision medicine is poised to profoundly transform therapeutic strategies via enhanced personalization based on individual biological, environmental, and clinical variables.

## Introduction

Precision medicine marks an important advancement in medical care, moving from conventional, uniform approaches to customized interventions informed by individual characteristics. This approach uses diverse biological datasets that reflect individual variations in genes, function, and environment to develop and refine procedures for therapeutic interventions and prognosis. This method aims to enhance treatment effectiveness and minimize adverse effects by stratifying patients according to response likelihood or disease risk (1). Precision medicine has evolved from a theoretical framework into a practical methodology that influences clinical decision-making across specialties. This evolution reflects a growing recognition that disease manifestations and treatment responses vary markedly among individuals owing to differences in genetic profiles, environmental exposures, and lifestyle factors. The foundational premise of precision medicine, which holds that healthcare interventions can be tailored to individual characteristics, has been established for decades; however, technological limitations have historically hindered practical implementation (2–4). The precision medicine movement emerged in the late 20th century and was significantly influenced by the Human Genome Project, which produced the first comprehensive map of the human genome and paved the way for progress in genomic technologies. This milestone triggered a series of technological advancements in genomic sequencing, biomarker identification, and molecular diagnostics, which increasingly broadened the clinical application of precision medicine. Initial implementation focused on oncology, where molecular profiling of tumors identified specific genetic alterations that could inform targeted therapy choices. Genomics initially drove the advancement of precision medicine; however, the field has progressively integrated different molecular aspects, including the transcriptome, proteome, metabolome, and microbiome (5, 6). This multi-omics approach enables comprehensive characterization of biological states and disease mechanisms. By incorporating environmental exposures, lifestyle factors, and social determinants, it has improved the precision medicine framework, acknowledging that health outcomes arise from complex interactions between genetic predispositions and environmental influences. The rapid increase in biomedical data, including proteomics, genomics, metabolomics, and digital health information, presents both opportunities and challenges for implementing precision medicine in clinical practice (7, 8).

Artificial intelligence (AI) is a key technology of this revolution, providing computational frameworks that can identify patterns and generate insights from vast and intricate biomedical data (9). The use of AI in precision medicine has significantly grown in recent years, fueled by rapid progress in computing capabilities and the abundance of large biological and clinical datasets. AI, including machine learning (ML) and deep learning (DL) approaches, can detect patterns and associations within diverse biological and clinical datasets that are often overlooked by conventional analytical techniques (9, 10). The shift toward AI has enabled researchers and clinicians to move from general disease classifications to a more detailed understanding of individual disease manifestations and therapeutic responses. Researchers in the field assert that “there is no precision medicine without AI,” underscoring that the computational requirements for analyzing multidimensional datasets demand substantial computing power and self-learning algorithms inherent to contemporary AI methodologies. These capabilities make AI a crucial tool throughout the precision medicine continuum, encompassing molecular characterization, disease risk prediction, treatment selection, and patient monitoring. This review presents a detailed analysis of the current applications, validation status, challenges, and future developments of AI in precision medicine (10, 11). Throughout this study, we have highlighted AI tools and platforms that are clinically useful or widely used in precision medicine. AI examples were selected based on their importance in recent research, clinically oriented with some validation and regulatory credibility.

## The role of AI in personalized treatment

AI is transforming precision medicine by analyzing large, diverse patient data, including genetics, clinical histories, imaging, and lifestyle factors. This analysis enables the detection of complexities and patient subgroups, supporting personalized treatment plans. Advanced Machine Learning (ML) and Deep Learning (DL) techniques boost diagnostic precision and facilitate early disease detection. AI algorithms can predict individual responses to treatments, helping to select drugs more accurately and reduce side effects by linking genetic or biomarker data with specific therapies. In addition, AI aids in discovering new disease biomarkers and accelerates drug development by modeling molecular interactions and enhancing clinical trial design via smart patient stratification (4, 12).

**Figure 1:**
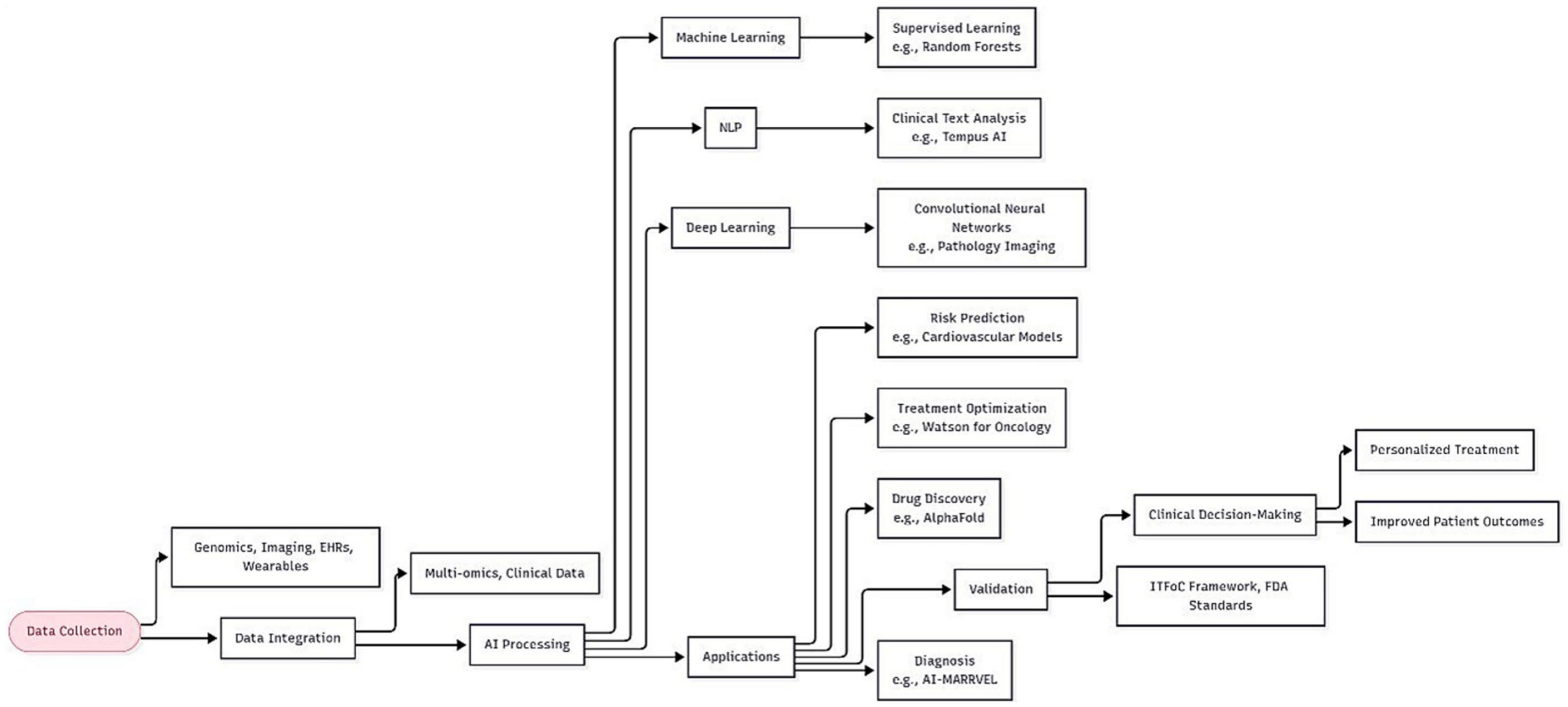
Workflow of artificial intelligence (AI) in precision medicine. This flowchart illustrates the integration of multi-omics data, AI methodologies (machine learning, deep learning, and natural language processing), and validation steps leading to personalized treatment decisions.

Several AI methodologies have proven effective in precision medicine. ML techniques—including supervised methods such as random forests and support vector machines, unsupervised approaches such as principal component analysis and clustering, and reinforcement learning—support predictive modeling, patient stratification, and adaptive therapies. Recurrent neural networks and convolutional neural networks (CNNs) have revolutionized medical imaging analysis and temporal health data interpretation. Natural language processing (NLP) helps extract clinically relevant information from unstructured medical texts, while knowledge representation techniques enable integration of domain knowledge with data-driven insights. These capabilities promote advanced personalization in healthcare, from preventive care to chronic disease management. Continuous analysis of patient data and comparison of outcomes across similar cases allow AI systems to optimize therapeutic strategies and update recommendations as new evidence emerges (10, 13–15). The AI workflow in precision medicine, from data collection to clinical decision-making, is summarized in Figure 1.

Recent advances in evolutionary algorithms, neural network designs, and other AI techniques demonstrate strong predictive power for complex clinical issues that conventional methods struggle to address. These technologies are particularly useful in oncology and cardiovascular medicine, providing impressive accuracy in predicting disease risk, treatment responses, and outcomes by analyzing multidimensional patient data (15).

## Application of AI in precision medicine and personalized treatment

In genomic medicine, AI addresses the challenge of interpreting extensive genetic information by prioritizing pathogenic variants and improving diagnoses. Systems such as Baylor Genetics’ AI-MARRVEL use ML to analyze exome sequences and clinical symptoms, thereby enhancing diagnostic accuracy for rare Mendelian disorders and reducing prolonged diagnostic journeys. The system has been trained on 3.5 million variants from documented cases (16). AI improves medical imaging and pathology by using DL algorithms that identify subtle irregularities in radiology scans and histopathology slides, outperforming humans in sensitivity and efficiency. In addition to diagnostics, AI permits dynamic predictions of disease risk by synthesizing factors such as genes, the environment, and lifestyle. Risk models for cardiovascular disease and diabetes outperform conventional calculators by identifying patterns in biomarkers, allowing preemptive interventions. AI enhances cancer risk stratification, enabling personalized screening protocols that optimize early detection and reduce unnecessary procedures (17–19).

AI transforms drug discovery and treatment optimization by enhancing pharmaceutical pipelines. ML identifies drug prospects, estimates toxicity, and repurposes existing treatments via effective chemical library screening and lead compound optimization, exemplified by the computational analysis that led to the identification of baricitinib for the treatment of COVID-19 (20). The Tempus AI TIME program exemplifies the strength of AI in improving clinical trials, having performed over 280 million searches across 94 sites, including 840,523 patients, within a 6-month period. This effort yielded 847,689 potential trial matches and resulted in 71 trial activations, considerably reducing activation times (21). Oncology treatment selection is enhanced by AI models that link tumor genetics to therapeutic responses, recommending regimens that optimize efficacy while minimizing toxicity. AI helps dosing personalization by analyzing pharmacokinetic variability, thereby ensuring therapeutic accuracy for medications with narrow efficacy windows. AI-driven wearables and remote sensors improve patient monitoring by identifying early indicators of clinical deterioration in chronic diseases or postoperative contexts. Predictive algorithms in heart failure management can preempt hospitalization by identifying trends in fluid retention, whereas continuous glucose monitoring in diabetes predicts hypoglycemic events, facilitating timely interventions. DL algorithms in diagnostics, trained on extensive annotated image datasets, can detect subtle abnormalities across various imaging modalities. In pathology, ML improves specimen evaluation by automating classification and integrating molecular data with conventional histopathology (22–24).

Nonetheless, AI integration presents significant challenges, including data privacy concerns, algorithmic bias, and the opacity (“black-box”) of sophisticated models. The World Health Organization (WHO) emphasizes the need for robust regulatory frameworks to ensure transparency, validation, and equitable implementation. WHO guidelines prioritize documenting AI limitations, implementing risk management throughout development lifecycles, and conducting rigorous clinical validation to address biases in training data. These measures aim to reconcile innovation with patient safety while addressing disparities intensified by biased algorithms. As AI advances alongside technologies such as CRISPR/Cas and multi-omics, its role in facilitating proactive, patient-centered care will grow. Multidisciplinary collaboration among clinicians, data scientists, and policymakers is essential for the responsible harnessing of AI’s potential, ensuring that advances in precision medicine are achieved equitably and ethically (25–27) (Tables 1–3).

**Table 1:** AI and its purpose and application in genomics and precision medicine.

AI name	Purpose	Application	Reference
AlphaFold	Protein structure prediction	Predicts 3D protein structures from amino acid sequences	(94)
Tempus AI	Clinical Trial matching/precision medicine	Algorithmic patient matching using EHR, NLP, and genomic data	(95)
Benevolent AI	Drug repurposing	Matches existing drugs to new indicators using biomedical data analysis	(96)
DeepVariant	Genomic variant calling	Initially for short-read data, new versions support long-read data like PacBio and ONT	(29, 97)
DeepTrio	Genomic variant calling	Incorporates three data types for short and long read; ideal for rare genetic diseases	(29, 98)
DNAscope	Genomic variant calling	Detects SNPs/InDels in data from PacBio/ONT	(29, 99)
HELLO	Genomic variant calling/Hybrid variant calling	Combines short/long-read sequencing data for improved accuracy	(29, 100)
Clair3	Genomic variant calling/Long-read analysis	Detects SNPs/InDels in data from PacBio/ONT	(29, 101)
PolyPhen-2	Genomics	Predicts impact of amino acid substitutions on protein function	(102)
DRAGEN (Illumina)	Genomics	Accelerated genomic data processing and variant calling	(103)
NLP-based Clinical Recommendation Tools	Oncology/Genomics	Literature mining for evidence-based recommendations	(69, 104)
Watson for Genomics (IBM)	Oncology	Interprets genomic data for cancer treatment recommendations	(105, 106)

**Table 2:** AI and its purpose and application in oncology and pathology.

AI name	Purpose	Application	Reference
Path AI	Digital pathology	Analyzes tissue biopsies for cancer diagnostics	(107, 108)
Princess Máxima AI	Paediatric oncology	Personalizes treatment for children unresponsive to standard therapy	(109, 110)
ITFoC AI Framework	Oncology (TNBC)	Predicts treatment response using real-world and molecular -omics data	(111)
MSK IMPACT	Oncology (tumor profiling)	Analyzes genetic makeup of tumors; mutation analysis	(112, 113)
OncoKB	Oncology	Interprets MSK-IMPACT results; Precision oncology knowledge base	(112, 114)
Watson for Oncology (IBM)	Oncology	Watson for Oncology (IBM)	(106, 115)
Foundation Medicine AI	Oncology	Genomic profiling and therapy matching	(116)
Google DeepMind Health	Ophthalmology, Oncology	Cancer detection from images	(117)

**Table 3:** AI and its purpose and application in cardiology, neurology, and other specialties.

AI name	Purpose	Application	Reference
Cardiogram AI	Cardiology	Detects arrhythmias and predicts heart disease from wearable data	(118, 119)
HeartFlow FFRct	Cardiology	Non-invasive coronary artery disease assessment from CT images	(120)
BioMind	Neurology/Pathology	Brain tumor and stroke diagnosis from imaging	(121)
Pharma AI (Insilico)	Target identification & molecule design	Integrates NLP, GANs, and reinforcement learning for multi-omics data analysis.	(122)
Enlitic	Radiology	AI for radiology image triage and diagnosis	(123)
NVIDIA Clara	Medical Imaging, Genomics	AI platform for imaging analysis and genomics	(124)
GNS Healthcare	Multiple (Oncology, Cardiology, etc.)	Causal ML for treatment optimization	(125)
Exomiser	Rare disease diagnostics	Prioritizes disease-causing variants in whole-exome or whole-genome sequencing data;	(57, 126)
AiCure	Clinical trial design	Monitor regular medication intake by schizophrenia patients (in phase-II trials)	(127, 128)
Recursion OS	Phenotypic drug discovery	Analyzes biological/chemical data with vision transformers (Phenom-2)	(129)
Gubra’s streaMLine	Peptide optimization	Combines high-throughput data with ML for multi-parameter optimization.	(130)
Bioptimus	Foundation models for biology	Trains universal AI models on multi-scale biological data	(131)
Iambic Therapeutics	Multi-scale drug design	Integrates Magnet (molecule generation) and NeuralPLexer	(132)
Melloddy	Federated learning	Collaborative ML across pharma datasets without data sharing	(133)
Digital Twins	Treatment simulation; Drug discovery	Virtual patient models for simulating interventions	(134)
TrialGPT	Clinical trial design	LLM for predicting patient eligibility and trial suitability	(135)

## AI in functional genomics

Next,-generation sequencing (NGS) technologies generate large datasets that pose significant computational challenges for quality control, alignment, and variant calling. AI methodologies have considerably improved the precision and efficacy of fundamental genomic analyses (28, 29). Nanopore sequencing technologies have witnessed tremendous progress with the use of recurrent neural networks that analyze variations in electrical impulses to identify altered nucleotides. This development augments the ability to detect epigenetic modifications, such as methylation, without the need for additional experimental procedures. The clinical interpretation of genomic variants is a key barrier to translating sequencing results into practical applications. AI systems have revolutionized this process by integrating diverse evidence sources and applying advanced predictive algorithms (29, 30). DL models have greatly improved the prediction of variant pathogenicity by incorporating functional genomics data, protein structural features, phenotypic correlations, and evolutionary conservation patterns. These approaches are particularly useful for comprehending splicing changes, noncoding regulatory regions, and missense variants as the functional impact cannot be inferred from sequence changes alone. Both commercial and academic platforms employ ML frameworks to automate parts of variant interpretation following professional guidelines, such as those from the American College of Medical Genetics and Genomics. These systems help standardize evidence evaluation, promote consistency among analysts, and reduce interpretation time, all while maintaining a high level of agreement with expert assessments (31–33). AI methodologies have transformed protein structure prediction and functional annotation, as exemplified by DeepMind’s AlphaFold system. AlphaFold uses DL to predict 3D protein structures with remarkable precision, achieving results comparable to those of experimental techniques such as X-ray crystallography for many proteins. This capability significantly impacts precision medicine as it permits structural assessment of disease-associated variants. Furthermore, it supports rational drug design that targets specific variants and enables the characterization of protein–protein interactions influenced by genomic alterations (34, 35). The technological advancements in AI have improved the functionality of variant databases, such as ClinVar, by enhancing variant classification, resolving interpretation inconsistencies, and extracting actionable insights from the available evidence (36, 37). ML methods support the automatic harmonization of variant classifications across different submitters by identifying patterns in their varying interpretations and suggesting consensus classifications based on the available evidence. NLP techniques enable the extraction of structured data from free-text explanations, making it easier to compare the evidence factors behind different interpretations. AI tools assist in proactive variant reclassification by continuously monitoring emerging evidence and flagging variants that might need reassessment as new data becomes available. This function is especially useful for variants initially labeled as variants of uncertain significance, many of which can be reclassified with advancements in technological capabilities and scientific understanding (38–41).

## AI in drug discovery

AI is transforming drug discovery by addressing inefficiencies in conventional pharmaceutical processes, which often take over 10 years and cost approximately $2.6 billion per approved drug. ML models improve target identification by simulating molecular interactions and predicting binding affinities. For example, DeepMind’s AlphaFold has predicted 3D structures for over 200 million proteins, supporting the discovery of new drug targets for diseases such as malaria and leishmaniasis (42, 43). Generative adversarial networks (GANs) facilitate drug candidate design by analyzing bioactive molecule databases. Insilico Medicine used GANs to discover INS018_055, an antifibrotic drug in Phase II trials, shortening the discovery time from 6 years to 18 months. Furthermore, AI aids in drug repurposing; for example, during the COVID-19 pandemic, BenevolentAI reviewed numerous biomedical papers and clinical trial data, identifying baricitinib as a promising candidate. Its dual mechanism—blocking viral entry via AP2-associated protein kinase 1 and reducing cytokine storms via JAK–STAT pathways—was confirmed in clinical trials. AI enhances clinical trial efficiency by accurately matching patients to studies. Nevertheless, challenges persist as ML models need large, high-quality datasets, and biases, such as the under-representation of non-European groups, limit their use. AI platforms, such as Atomwise, which screens 3 trillion virtual compounds weekly, highlight the move toward data-driven, cost-effective drug development despite these obstacles (44, 45).

## AI in treatment optimization

**Figure 2:**
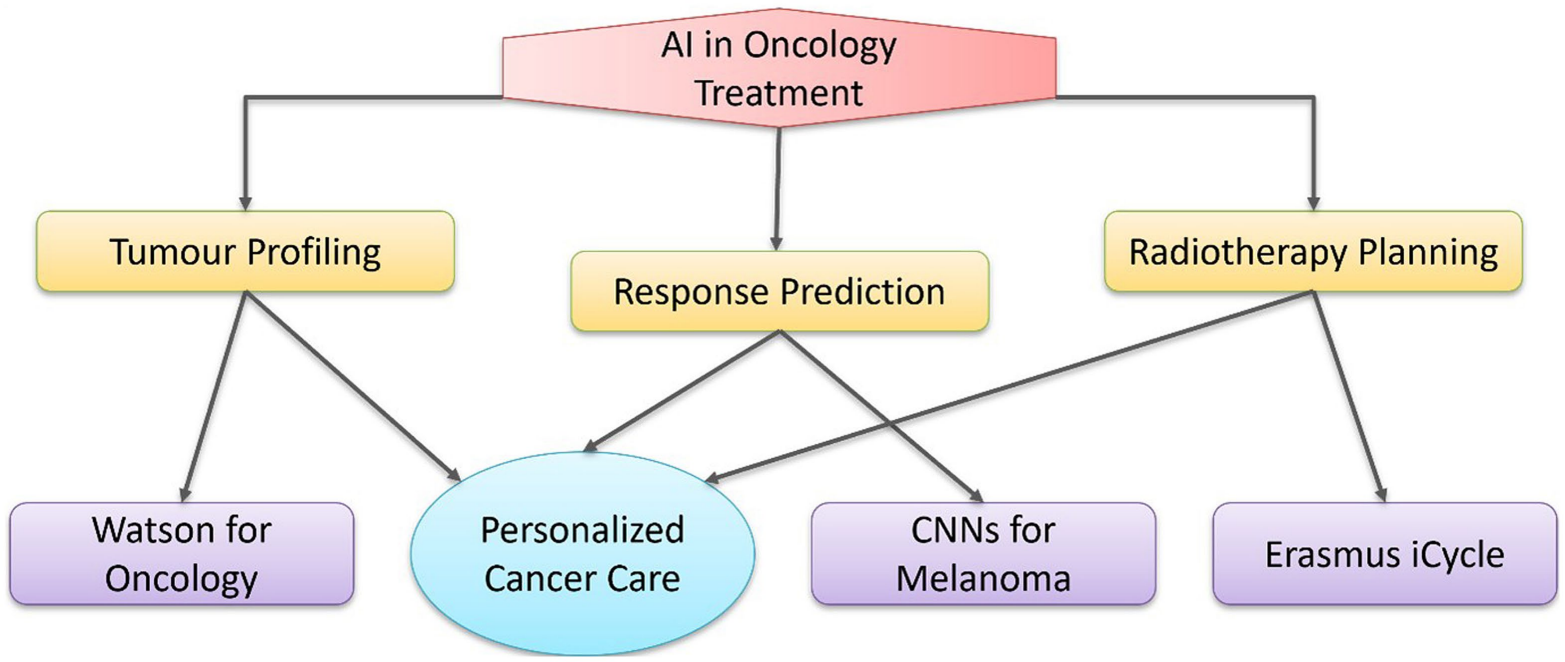
AI applications in oncology and precision oncology treatment optimization. This diagram illustrates how AI enables personalized cancer care via tumor profiling (e.g., Watson for Oncology), response prediction (e.g., convolutional neural networks for melanoma), and radiotherapy planning (e.g., Erasmus iCycle).

AI enhances therapy optimization by synthesizing multimodal data, thereby enabling the provision of therapies tailored to specific patients. In oncology, tools such as IBM Watson for Oncology analyze cancer genetics, treatment histories, and results from multiple cases to recommend therapy protocols (46). In radiotherapy, AI technologies such as Erasmus I-cycle automate treatment planning via multicriteria optimization, reducing planning time from days to hours while preserving dosimetric accuracy (47). ML models, including CNNs, use predictive analytics to predict treatment responses from multi-omics data. Liu et al. developed a regression-based algorithm to predict PD-1 inhibitor susceptibility in patients with melanoma by combining genetic and clinical data (48). Johannet et al. (49) went a step further and trained CNNs on histopathology slides to accurately predict immunotherapy responses. AI is enhancing cancer treatment by expediting drug development, matching patients with clinical trials via biomarker analysis, and tailoring drug delivery. ML and neural networks can predict drug reactions and improve treatment protocols, resulting in more efficient and personalized cancer care (50). Explainable AI approaches have been used in acute myeloid leukemia to correlate drug screening outcomes with genetic events, facilitating predictions of patient responses to specific medications and aiding in the formulation of effective, accessible treatment plans (51). AI improves medication dosing by using digital twins—virtual patient models that simulate therapies. These tools, evaluated in heart disease and diabetes management, predict hemodynamic responses to pharmacologic treatments or insulin protocols, facilitating proactive modifications (52–54). Moreover, AI might enhance therapeutic protocols for chronic conditions such as heart failure by predicting the likelihood of decompensation using electronic health records (EHRs) and wearable sensor data, thereby facilitating proactive interventions (55). Interoperability issues between EHR systems and the opacity of AI algorithms erode clinician trust. The advancement of AI, along with the incorporation of real-world data from wearables and patient-reported outcomes, will improve the customization of care (9). The integration of AI applications into oncology treatment optimization, including tumor profiling, response prediction, and radiotherapy planning, is depicted in Figure 2.

## AI in rare disease diagnostics

In genomics, AI-driven technologies such as DeepVariant and Phen-Gen analyze next-generation sequencing data, linking genetic variants with clinical phenotypes to prioritize potential disease-causing genes. For instance, Phen-Gen integrates a patient’s genomic and phenotypic data with AI-driven imaging, improving diagnostic accuracy (52% more accurate gene prediction). Exomiser and similar tools combine genomic and phenotypic data to prioritize pathogenic mutations, thereby shortening diagnostic timelines from years to weeks (56, 57). In medical imaging, DL models such as DeepGestalt (Face2Gene platform) analyze facial images to detect unique patterns associated with >200 rare genetic diseases, including Cornelia de Lange and Monogenic Rare Diseases, with 91% accuracy in the top-10 diagnoses. In addition, next-generation phenotyping technologies powered by AI (such as Eye2Gene) extend this approach to retinal images, accelerating and improving the accuracy of diagnoses for diseases with distinct physical manifestations. NLP algorithms enhance rare disease diagnosis by extracting essential information from EHRs and physician notes, identifying atypical symptom combinations that may suggest a rare disorder. Beyond diagnosis, graph neural network models such as TxGNN are revealing novel therapies for uncommon and ultra-rare diseases via drug repurposing, immensely expanding the therapeutic options. Together, these AI technologies are helping healthcare practitioners overcome data scarcity, minimize diagnostic delays, lower healthcare costs, and provide rapid, personalized insights, advancing rare disease diagnosis and management (58, 59).

## AI and CRISPR in precision medicine

ML systems such as DeepCRISPR predict optimal guide RNA sequences and minimize unexpected genomic changes, boosting the safety and effectiveness of CRISPR-based cancer therapies. By analyzing large genomic datasets, DeepCRISPR accurately detects potential off-target sites, reducing unintended genetic edits. In CAR-T cell therapies, AI models enhance CRISPR editing to disable inhibitory genes such as PD-1 and stimulate NK cells, increasing T-cell activity against tumors and identifying the best targets for immune checkpoint blockade. In drug resistance research, AI pinpoints CRISPR targets that make cancer cells more sensitive to chemotherapy—for example, AI-led removal of MDR1/ABCB1 genes significantly improves doxorubicin sensitivity in breast and colon cancers. Furthermore, AI improves gRNA design by evaluating factors such as sequence composition and chromatin accessibility, enabling more efficient gene editing (60–62). Diagnostic solutions such as SHERLOCK and DETECTR use AI algorithms in conjunction with Cas12/Cas13 systems to detect cancer-specific mutations in liquid biopsies, offering high sensitivity and facilitating early detection and treatment assessment. AI expedites the design of personalized therapies by analyzing patient genetic data to determine which oncogenes (such as KRAS, MYC, and EGFR) should be targeted with CRISPR for better treatment outcomes. These AI-designed CRISPR strategies are often combined with immunotherapy. ML models identify the most effective gene editing combinations to prevent tumors from evading the immune system. The integration of AI and CRISPR is transforming cancer treatment from broad, cytotoxic approaches to precise, personalized therapies that target genetic susceptibilities while minimizing damage to healthy cells (61–63).

## Validation status of AI tools in precision medicine

As AI technologies advance from research to clinical application, it is essential to understand how well-validated AI tools perform in precision medicine. Although AI has immense potential to personalize diagnostics and treatments for individual cases, the absence of comprehensive, standardized validation processes impedes its broad clinical implementation. The European Information Technology for the Future of Cancer consortium has established a comprehensive framework for validating AI in oncology, highlighting seven essential actions: defining intended use, dataset selection, data safety (encompassing privacy, quality, and security), target population, evaluation timing, performance metrics, and explainability. This paradigm aims to ensure that AI technologies are rigorously, impartially, and transparently evaluated before clinical deployment, using external real-world datasets to benchmark prediction efficacy and safety.

An assessment of FDA-regulated imaging-based AI algorithms revealed substantial deficiencies in validation methodologies. Of the 118 AI/ML algorithms examined, 17 lacked published validation claims or data and only nine used validation datasets comprising >1,000 patients. The lack of transparency and inadequate dataset sizes hinder the evaluation of generalizability and potential biases, raising questions about the justification for clinical use (64). The lack of transparency in the design and validation may be attributed to the insufficiency in the dataset details, external validation and patient’s diversity. FDA has approved several medical devices that use AI and ML, including IDx-DR for diabetic retinopathy diagnosis. IDx-DR exhibited approximately 90% sensitivity and specificity in identifying retinopathy across various populations; however, its implementation is limited to high-resource settings because of associated infrastructure costs (65, 66). Moreover, although device approval often depends on technical precision, it does not ensure clinical efficacy or improved patient outcomes. Authentic clinical validation requires external evaluation in real-world settings and, preferably, randomized clinical trials to demonstrate effects on patient care (67). Insurance coverage determinations depend on evidence showing that AI use leads to improved outcomes, underscoring the need for thorough validation beyond regulatory endorsement.

Initiatives to build local validation infrastructures are underway, especially in imaging, where institutions are urged to evaluate AI models on their own patient populations before incorporating them into clinical procedures. These infrastructures are crucial for ensuring accuracy, generalizability, and health equity, yet they encounter obstacles related to data privacy, intellectual property, and model diversity (68). The incorporation of AI in cancer genomics and other domains is increasing, although obstacles remain regarding data prerequisites, algorithmic openness, platform standardization complications, reproducibility, difficulties in clinical workflows and real-world evaluation (69). Although AI technologies have potential in precision medicine, their clinical implementation is hindered by inconsistent and often inadequate validation procedures. Addressing these gaps via standardized frameworks, transparent reporting, and stringent external validation is crucial for the safe and effective deployment of AI in personalized healthcare (9, 67–70). To enhance the accuracy of AI tools, rigorous validation preferably involving at multi-site, prospective cohorts, external test sets, and, when applicable, randomized or guided trial designs are required. These stringent methods augment the accuracy of the AI tools and develop clinicians’ trust and support decision-making.

## Economic impact of AI in precision medicine

AI-driven precision oncology offers substantial economic advantages by facilitating early cancer diagnosis, enhancing treatment selection, and minimizing the use of ineffective medications, thereby saving healthcare expenditures and improving patient outcomes. AI-driven analysis of NGS data can detect actionable mutations and predict drug resistance, enabling doctors to customize medications and prevent unnecessary treatments, thereby reducing resource waste and adverse effects. Systems such as IBM Watson for Oncology facilitate clinical decision-making by rapidly analyzing complex patient data to suggest cost-effective, evidence-based treatments, resulting in a more efficient allocation of healthcare resources (71–73). AI accelerates medication discovery and repurposing, reducing the time and costs involved in introducing new therapeutics to the market. In pediatric oncology, AI improves risk classification and personalized care, thereby enhancing survival rates and alleviating the cost burden of extended or unsuccessful therapies (73, 74). Furthermore, AI-powered digital pathology and imaging streamline diagnostic processes, enhancing precision and efficiency while reducing labor costs, making them particularly beneficial in resource-constrained environments. In low- and middle-income countries, implementation faces excessive infrastructure costs, limited workforce, and non-robust digital health systems, requiring financing and strategies for improving skill. Nonetheless, reaping economic benefits requires investment in robust data infrastructure, legislative adjustments, and interdisciplinary collaboration to ensure equitable access and integration into clinical practice. AI precision oncology illustrates how sophisticated analytics can revolutionize the economics of cancer care by optimizing treatment efficacy and reducing unnecessary costs via personalized, data-driven interventions (75–77).

## Challenges and ethical considerations

**Figure 3:**
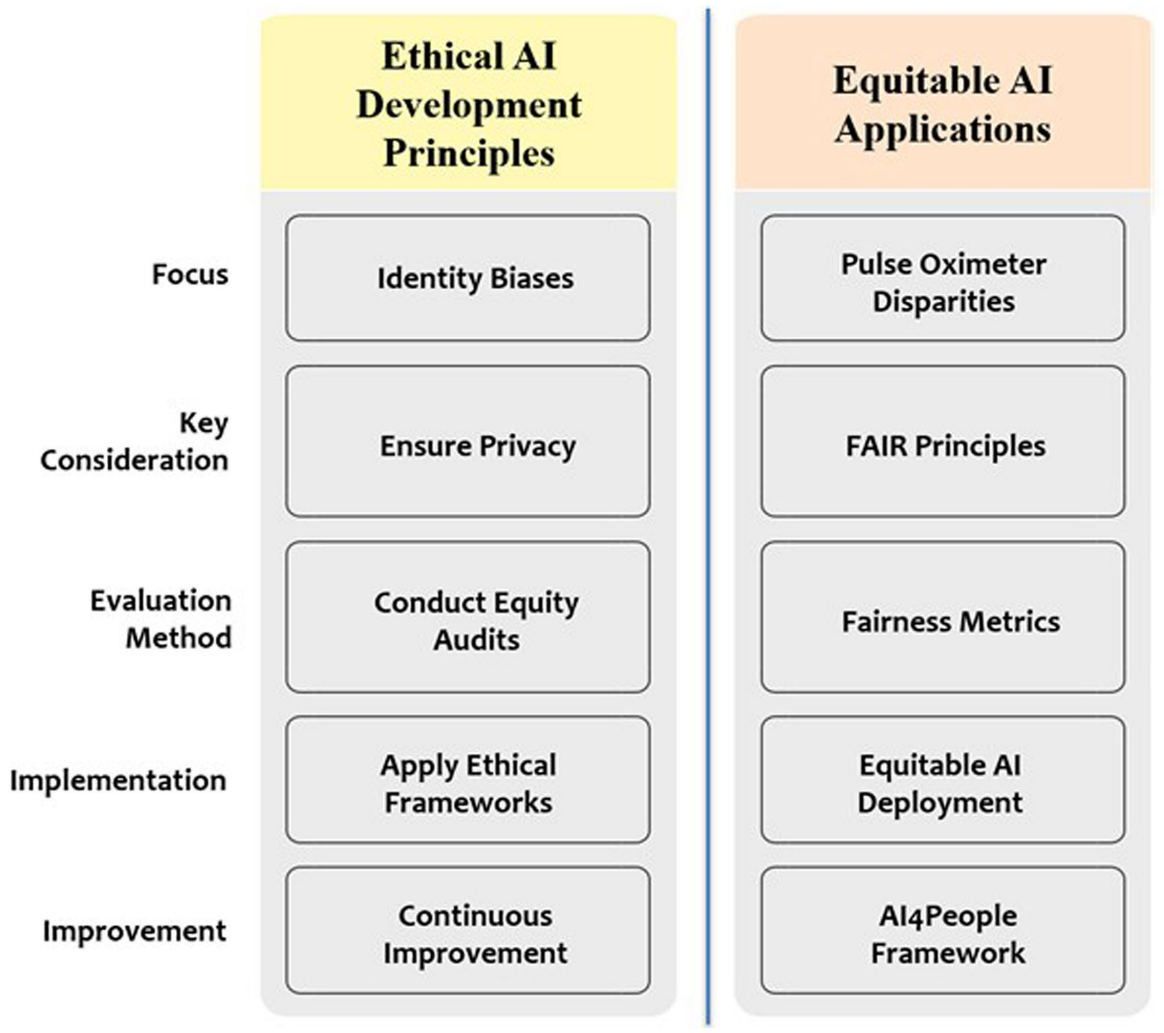
Ethical considerations in the use of artificial intelligence (AI) for precision medicine. This diagram illustrates the iterative process of ensuring ethical AI use by identifying biases (e.g., pulse oximeter disparities), ensuring privacy (e.g., FAIR principles), conducting equity audits, and applying ethical frameworks (e.g., AI4People), leading to equitable AI deployment.

The integration of AI into precision medicine raises significant ethical and practical challenges. Data privacy risks are substantial; genomic data, even when anonymized, may be reidentified via public databases. Algorithmic bias contributes to healthcare disparities; for example, pulse oximeters tend to miscalculate oxygen saturation in individuals with darker skin because training data are predominantly based on lighter skin tones. This discrepancy results in delayed COVID-19 treatment for Black and Hispanic populations (78–80). Cardiovascular risk models built with EHRs often poorly reflect socioeconomically disadvantaged groups, missing 30% of high-risk Black patients. Moreover, informed consent processes struggle with the lack of transparency in AI (81). Ethical dilemmas arise in resource allocation, as evidenced by AI tools that prioritized ICU beds during COVID-19, which disadvantaged patients with disabilities owing to biased outcome metrics (82). Furthermore, data quality challenges within EHRs due to unclear terminologies, incomplete entries and varied data formats affects the clinical decision making. These challenges may be overcome by developing standardized data formats, data pipelines and ontology mapping. From a regulatory point of view, the multiple stakeholder’s collaborations comprising developers, regulators and clinical institutions should come to forefront for innovations and safety in AI supported medical devices. Implementing FAIR principles (Findable, Accessible, Interoperable, and Reusable) in health data and conducting equity audits for AI models can help tackle these challenges (83, 84). Initiatives such as AI4People propose ethical frameworks that focus on transparency, justice, and accountability, but their implementation is still lacking. Collaborative efforts such as the NIH Bridge2AI program aim to democratize data access; however, systemic inequalities in digital literacy and infrastructure persist, particularly in low-income regions (85–87). Figure 3 depicts the process of addressing ethical challenges in AI for precision medicine, including bias mitigation and privacy protection.

## Conclusion and future directions

AI is dramatically changing precision medicine by enabling the incorporation and analysis of extensive, complex information, including genomes, medical imaging, EHRs, and real-time biological data. This convergence allows doctors to provide tailored care, recognize patient subgroups with distinct treatment responses, and enhance diagnostic and prognostic accuracy. AI-driven techniques are already demonstrating utility in biomarker identification, tumor growth prediction, and patient stratification for clinical trials, accelerating the conversion of massive data into practical clinical insights. Despite these advances, fewer than 5% of AI technologies achieve widespread clinical use because of fragmented validation requirements and limited real-world data. Collaborative initiatives, exemplified by the EU’s *In Silico* World project, aim to standardize validation processes globally; yet, persistent issues in data management and clinician trust remain. As legal frameworks progress, emphasis should shift from discrete validation to comprehensive lifecycle assessment, ensuring that AI solutions adapt safely to evolving healthcare environments (88, 89).

The future of AI in precision healthcare will be shaped by three critical trends. The use of sophisticated ML and DL models will continue to grow, especially in genomics, digital pathology, and radiology, where AI can quickly analyze high-dimensional data and reveal new disease signatures. Developing reliable, explainable AI systems is crucial to ensure transparency, reproducibility, and clinician confidence in AI-assisted decision-making. Third, privacy-preserving technologies such as synthetic data generation and federated learning will be critical to enable secure, large-scale data exchange among institutions, which is vital for effective model training and validation. Federated learning promotes collaborative model training while maintaining the confidentiality of sensitive data, hence safeguarding privacy among institutions. Nonetheless, it encounters challenges such as considerable communication costs, issues related to data heterogeneity, and unclear regulatory frameworks. Regulatory frameworks and collaborative initiatives among stakeholders, including doctors, researchers, regulators, and industry, will be required to address challenges related to data quality, bias, and ethical considerations (90–93).

Numerous AI enterprises and platforms are making substantial advances in this domain. NVIDIA offers high-performance computing and AI medical cloud systems that permit rapid analysis of clinical and genomic data, advancing precision-based cancer genomics in clinical settings. Illumina’s DRAGEN platform combines AI and field-programmable gate arrays to expedite genomic data processing, facilitating faster, more precise variant calling for personalized medicine. In addition, enterprises such as Tempus and Foundation Medicine are integrating AI with multi-omics and clinical data to inform targeted cancer medicines and improve patient outcomes. As AI advances, collaboration across disciplines, including clinicians, data scientists, and policymakers, will be necessary to ensure that innovations prioritize patient welfare rather than commercial interests. The ethical and equity problems addressed in this review are fundamentally connected with the validation and reporting of AI tools. Biases resulting from limited or unrepresentative training and validation cohorts might lead to varying performance among demographic and socioeconomic groups, hence increasing existing gaps in healthcare. Thus, robust and open validation that incorporates various populations and real-world data is both a technological requirement and a fundamental approach for advancing fairness and equitable access in AI-driven precision medicine.

Embedding ethical principles into AI design and governance enables precision medicine to achieve its objective of providing equitable, proactive, and personalized care for all individuals.
